# P-510. Invasive Group B Streptococcus among hospitalized pregnant and postpartum women, 2016 – 2024: a United States database study

**DOI:** 10.1093/ofid/ofaf695.725

**Published:** 2026-01-11

**Authors:** Sarah Willis, Katharina Schley, Wencheng Zhu, Eileen M Dunne, Kyla hayford, Jennifer Moisi

**Affiliations:** Pfizer, Inc., Cambridge, MA; Pfizer Pharma GmbH, Berlin, Berlin, Germany; Genesis Research Group, Hoboken, NewJersey; Pfizer, Inc., Cambridge, MA; Pfizer, Toronto, Ontario, Canada; Pfizer Vaccines, Collegeville, Pennsylvania

## Abstract

**Background:**

*Group B Streptococcus* (GBS) is a leading cause of severe disease among pregnant individuals, neonates, and infants. Postpartum individuals with GBS are also at risk of endometritis and postoperative wound infections. In this study we describe demographics, clinical characteristics, and health care resource utilization among pregnant and postpartum women hospitalized with laboratory-confirmed invasive GBS (iGBS).

**Methods:**

Individuals ≥18 years of age who had a positive GBS specimen collected from a sterile site during an inpatient admission were identified in the PINC AI™ Healthcare Database, which includes ∼1200 US hospitals, between 2016-2024 (index admission). Female patients who had a diagnosis and/or procedure code associated with pregnancy or delivery 0-42 days prior to index admission were categorized as pregnant or postpartum. We characterized maternal age, race, payor, and underlying health conditions 0-365 days before index admission. We also captured hospital length of stay, intensive care unit (ICU) admissions during index admission, and 90-day readmissions.

**Results:**

We identified 150 pregnant and postpartum women hospitalized with iGBS between 2016-2024. Most women (81%) gave birth during the index admission, 8% were postpartum, and 11% were currently pregnant. Average age at admission was 29.4 years (standard deviation [SD] = 6.6 years). Half of women (53%) were White, 33% Black, 2% Asian, and 13% reported other or unknown race. Half of women (52%) were covered by commercial payors and 44% were Medicaid beneficiaries. The most common underlying health conditions were immune suppression (23%), obesity (15%), asthma (8%), and smoking (7%). The average length of the index admission was 5.2 days (SD = 5.0 days) and 13% of women were admitted to the ICU. In addition, 15% were readmitted to the same hospital < 90 days after discharge.

**Conclusion:**

These analyses provide valuable information on iGBS disease during pregnancy and the post-partum period, and patient characteristics and health care utilization associated with this infection. Future analyses should focus on estimating disease incidence and assessing predictors of severe disease among women with iGBS to guide strategies for disease prevention.

**Disclosures:**

Sarah Willis, PhD, Pfizer, Inc.: Employee|Pfizer, Inc.: Stocks/Bonds (Public Company) Katharina Schley, Dr. rer pol., Pfizer: Employee|Pfizer: Stocks/Bonds (Private Company) Eileen M. Dunne, PhD, Pfizer: Employee|Pfizer: Stocks/Bonds (Public Company) Kyla hayford, PhD, Pfizer: Stocks/Bonds (Public Company) Jennifer Moisi, PhD, Pfizer Vaccines: Employer|Pfizer Vaccines: Stocks/Bonds (Public Company)

